# Ketamine Implicated in New Onset Seizure

**DOI:** 10.5811/cpcem.2019.9.44271

**Published:** 2019-10-21

**Authors:** Christopher W. Meaden, Stacey Barnes

**Affiliations:** St. Joseph’s University Medical Center, Department of Emergency Medicine, Paterson, New Jersey

## Abstract

Ketamine is used widely in emergency departments for a variety of purposes, including procedural sedation and pain management. A major benefit of using ketamine is the rapid onset and lack of respiratory depression. The known side effects include emergence reactions, hallucinations, hypertension, dizziness, nausea, and vomiting. Recent studies have shown the benefit of ketamine for refractory status epilepticus; however, this application of the drug is still being studied. We present a case where ketamine likely induced a seizure in a patient on whom it was used as a single agent in procedural sedation. Seizure is not a known side effect of ketamine in patients without a seizure history. Given the eagerness over additional uses for ketamine, this novel case of a seizure following procedural sedation with ketamine should be of interest to emergency providers.

## INTRODUCTION

Ketamine is a versatile and effective drug used commonly in the emergency department (ED) to aid in the treatment of various pathologies. Although it is now commonly administered in EDs, it was first made available for commercial use in 1970. The initial use of ketamine was as a rapid-acting, dissociating anesthetic.^1^ Ketamine, a derivative of phencyclidine, was initially created to reduce the adverse psychotomimetic effect and lessen the abuse potential when compared to its parent drug. Because of its effect profile, phencyclidine was removed from the market in 1978.^2^ Ketamine is desirable for anesthetic use in procedural sedations, due to its rapid onset, relatively short half-life, and lack of respiratory depression.^3^

Although initially used as an anesthetic, ketamine has had evolving implications for other therapeutic uses. Dosing and route of administration vary for its evolving clinical uses; however, a dose of 1–2 milligrams per kilogram (mg/kg) is typically administered intravenously (IV) during procedural sedations. Ketamine causes a dissociative sedation, defined as sedation without a complete loss of consciousness but associated with a catatonic state, as well as amnesia. The drug has been used for procedural sedation, treatment of agitated delirium, analgesia and anti-inflammatory effects, treatment of depression and schizophrenia, and more recently for refractory status epilepticus^3^,^4^; however, like other medications, it is not without side effects. Adverse effects of ketamine that have been well described include the following: psychoactive effects such as hallucinations; visual disturbances including diplopia and ocular nystagmus; potential for abuse; emergence reactions as the medication wears off; dizziness; and nausea and vomiting.^3^

Despite reports of recent uses of ketamine for the treatment of seizures, we describe a case where a 15-year-old autistic girl, without a prior seizure history, received ketamine for a procedural sedation and had a new onset seizure during the process with a prolonged postictal period – a previously unreported adverse effect of the drug.

## CASE REPORT

A 15-year-old female with a past medical history of only autism spectrum disorder presented to the ED with complaints of a laceration to her right fourth digit and an abrasion to her right third digit, which were sustained approximately 22 hours prior to ED arrival. Before presenting to the ED, the patient’s mother attempted wound care at home; however, secondary to persistent bleeding, the patient was brought to a referral urgent care center that then subsequently sent the patient for evaluation at our ED.

On evaluation in the ED, the patient was at her baseline mental status as per her parents. Physical examination revealed a weight of 53 kg, a temporal temperature of 37 degrees Celsius, a heart rate of 71 beats per minutes, a respiratory rate of 18 breaths per minute, a blood pressure of 114/86 millimeters of mercury, and a room air oxygen saturation of 99%. She was noted to have a small abrasion to the medial aspect of the distal phalanx of the right third finger and a one-centimeter, elliptical-shaped laceration to the medial aspect of the distal right fourth finger that was actively bleeding. She had full range of motion of all 10 of her digits with a capillary refill of less than two seconds on each digit. Of note, her neurologic exam revealed the patient to be at her baseline status as per her parents, awake and alert, and she was symmetrically moving all of her extremities equally. The remainder of her examination was normal.

Due to continued bleeding of the laceration despite other attempts at hemostasis, and after a discussion with the patient’s parents regarding increased risk of infection because the wound had been open for 22 hours, they consented to have the laceration repaired with sutures in the ED. Because of the patient’s baseline mental status, secondary to her autism spectrum disorder, the decision was made to perform the laceration closure under procedural sedation.

For the procedural sedation, there was no IV access available; thus, the medication was administered intramuscularly. It had been greater than four hours since her last meal, she was able to fully flex and extend her neck, and she was able to fully open her mouth. We chose ketamine as the sedating agent for the procedure at a dose of 3–5 mg/kg, which is in the accepted intramuscular dosage range. Consent for the sedation was obtained from her parents after a full discussion regarding the risks and benefits of sedation and the known adverse effects of ketamine.

Ketamine was administered via a one-time dose intramuscularly at the start of the procedure; the total dose given was 150 mg, which was less than the recommended intramuscular dose. The patient was on a cardiac monitor with pulse oximetry and end-tidal carbon dioxide monitoring throughout the procedure and remained normoxic. The laceration on the right fourth digit was repaired using an aseptic technique after cleansing of the site, with four 4-0 chromic gut sutures. No local anesthetic was administered. As the last stitch was tied and nine minutes after ketamine was administered to the patient, she was observed to have generalized tonic-clonic seizure of one-minute duration that self-resolved without any administration of further medications. Medical toxicology was consulted and advised to evaluate the patient for a new onset seizure, as ketamine was not known to induce seizure activity.

The patient remained postictal in the ED, IV access was established, and a complete blood count, basic metabolic panel, and a computed tomography (CT) without contrast of the head were ordered. Blood analysis was noted to be within normal limits for our institution’s normal value range. The head CT was read by radiology to show mild colpocephaly of the ventricles with uncertain significance as there was a present corpus callosum and no evidence of transependymal resorption or other white matter findings.

**CPC-EM Capsule**

What do we already know about this clinical entity?

*Ketamine is a commonly administered sedative used in emergency departments for procedural sedation in pediatric patients, including those with autism spectrum disorder (ASD) and other developmental delays*.

What makes this presentation of disease reportable?

*We present a patient without a prior history of seizures, known to have ASD and undiagnosed colpocephaly who experienced a seizure after administration of intramuscular ketamine for laceration repair*.

What is the major learning point?

*Ketamine can have adverse effects such as inducing seizures in patients with undiagnosed colpocephaly or developmental delays as described in this case*.

How might this improve emergency medicine practice?

*Emergency physicians have multiple different options to consider for procedural sedation and should consider pros and cons to each agent prior to their administration*.

Pediatric neurology was consulted, and the case, laboratory findings, and imaging findings were discussed. Pediatric neurology recommended inpatient admission if the patient maintained altered mental status and to otherwise follow up with them for an electroencephalography (EEG) due to new onset seizure. The patient continued to be hemodynamically stable in the ED and maintain her airway, but did not return to her baseline and had one episode of vomiting despite observation for four hours after the observed seizure activity. At that time, she was admitted to the pediatric step-down unit for further evaluation and monitoring.

While in the pediatric step-down unit, the patient gradually returned to her baseline mental status; she had no further seizure activity throughout her stay and required no administration of any medications. She was evaluated by neurology and underwent a one-hour EEG, which showed no areas of focal slowing, epileptiform discharges, or electrographic seizures. The patient was discharged in stable condition after undergoing the EEG to outpatient follow-up on a course of cephalexin for empiric antibiotic coverage of her repaired finger laceration. The patient was then lost to follow-up.

## DISCUSSION

For nearly 50 years, ketamine has proven to be a safe anesthetic drug with potent analgesic properties and a wide range of uses in clinical practice.^2^ Emergency physicians have become comfortable with using this drug regularly, for both procedural sedation and management of pain. The benefits, as well as the known side effects, have been well documented. It is considered relatively safe when used with proper precautions in the ED and even in the prehospital setting and has been successfully administered to pediatric patients with autism spectrum disorder for routine ED procedures, such as laceration repair.^4^

One of the newer reported uses for ketamine is for the treatment of refractory status epilepticus. Researchers have shown that during prolonged seizures, the number of activated gamma-aminobutyric acid (GABA)-A receptors on the postsynaptic membrane gradually decreases, whereas the number of inactive GABA-A receptors increases.^5^,^6^ This causes a significant reduction in the efficacy of antiepileptic drugs that target the GABAergic system, including benzodiazepines, propofol, and phenobarbital. In contrast, the number and activities of N-methyl-D-aspartate (NDMA) receptors increases over time.^7^ Subsequently, a drug such as ketamine, which is a noncompetitive NDMA receptor antagonist, may play a role in treating status epilepticus. However, as this case clearly illustrates, ketamine may actually induce seizures in certain populations.

Ketamine use can cause excitatory effects on the central nervous system. The U.S. Food and Drug Administration already recommends that ketamine be contraindicated in patients with severe hypertension and allergies to ketamine. It should be used with caution in patients with coronary artery disease, heart failure, glaucoma, atherosclerosis, pulmonary heart disease, pulmonary hypertension, severe intracranial hypertension, pregnancy, history of mental illness, hyperthyroidism, tachyarrhythmia, adrenal pheochromocytoma, and alcoholism.^7^ Despite this extensive list of exclusions, there is no caution about administration of ketamine to patients with autism. Additionally, ketamine has been recommended by multiple studies, including in recent literature, as a procedural sedation agent in pediatric patients with autism spectrum disorder for use in laceration repair.^4^

There is no evidence that ketamine is likely to precipitate generalized convulsions, even in patients with both a history of epilepsy and an abnormal EEG.^8^ Convulsive activity and other deleterious neurologic sequelae may be seen in ketamine toxicity from overdose. Ketamine toxicity is more commonly seen when ingested recreationally, rather than in clinical practice. During recreational ingestion, ketamine may be laced with other contaminants and may not be a pure drug; thus, it is far from clear whether the clinical effects seen are due to ketamine or an adulterant found within an admix of a street formulation. Furthermore, what we know about ketamine toxicity and overdose is from animal literature and occurs at significantly higher doses than those administered to the patient in the case we have presented here.^9^

## CONCLUSION

Notwithstanding a lack of literature to support it, this case suggests that ketamine induced a seizure in a patient with no prior history of epilepsy. As more research emerges on the use of ketamine for refractory status epilepticus in addition to its other broad range of uses in the ED, perhaps the list of its side effects should be expanded more broadly. More research is needed regarding the administration of ketamine to patients with cognitive conditions, such as autism, and neuroanatomical abnormalities such as colpocephaly, as they may have a lower seizure threshold when ketamine is administered. Given the frequency with which ketamine is administered in EDs, emergency physicians should be aware that ketamine could potentially induce seizures.
